# An Imported Case of Gigantic Amoebic Liver Abscess in a 24-Year-Old Woman in Singapore

**DOI:** 10.1155/crdi/6230349

**Published:** 2025-03-20

**Authors:** Edwin Chong Yu Sng, Jean-Marc Chavatte

**Affiliations:** ^1^Department of Infectious Diseases, Changi General Hospital, Singapore, Singapore; ^2^National Public Health Laboratory, National Centre for Infectious Diseases, Ministry of Health, Singapore, Singapore

**Keywords:** amoebiasis, amoebic liver abscess, *Entamoeba histolytica*, imaging, polymerase chain reaction (PCR)

## Abstract

A liver abscess can be due to several different microbiological aetiologies. While pyogenic liver abscess is most frequently encountered, amoebic liver abscess and hydatid cyst caused by the parasites, *Entamoeba histolytica* and *Echinococcus granulosus,* respectively, should be considered whenever there is epidemiological exposure. As parasitic infections are now rarely seen in clinical practice in developed countries with improvement in sanitation, lack of clinical experience in managing amoebic liver abscesses and overlapping clinical and imaging features between amoebic and pyogenic liver abscesses may lead to delay in diagnosis. In particular, although amoebic liver abscesses respond well to treatment if diagnosed early, they can progress and rupture with high mortality if treatment is delayed. Hence, early diagnosis and prompt initiation of antimicrobials are crucial to prevent complications and death. This case report highlights a case of a very large 21 cm ALA in a young lady to illustrate the challenges faced during diagnostic evaluation.

## 1. Introduction

A liver abscess can be due to several different microbiological aetiologies. Pyogenic liver abscesses (PLAes) are due to bacterial infections and may be mono- or polymicrobial—*Escherichia coli* and *Klebsiella pneumoniae*are the most common pathogens. While PLA is most frequently encountered, amoebic liver abscess (ALA) and hydatid cyst caused by the parasites, *Entamoeba histolytica* and *Echinococcus granulosus,* respectively, should be considered whenever there is epidemiological exposure. Amoebiasis is distributed worldwide. Due to its faecal–oral route of transmission, the highest burden is in developing countries with poor sanitation. In comparison, amoebiasis is seldom encountered in clinical practice in developed countries and is diagnosed almost exclusively in immigrants from endemic areas or in returning travellers, among which *E. histolytica* is the third most frequently isolated pathogen in those with infectious gastroenteritis [1]. Lack of clinical experience in managing ALAes and overlapping clinical and imaging features between ALAes and PLAes may lead to delay in diagnosis. Although ALAes respond well to treatment if diagnosed early, they can progress and rupture with high mortality if treatment is delayed. Hence, early diagnosis and prompt initiation of antimicrobials are crucial to prevent complications and death. This case report highlights a case of a very large 21 cm ALA in a young lady to illustrate the challenges faced during diagnostic evaluation.

## 2. Case Presentation

**Figure 1:**
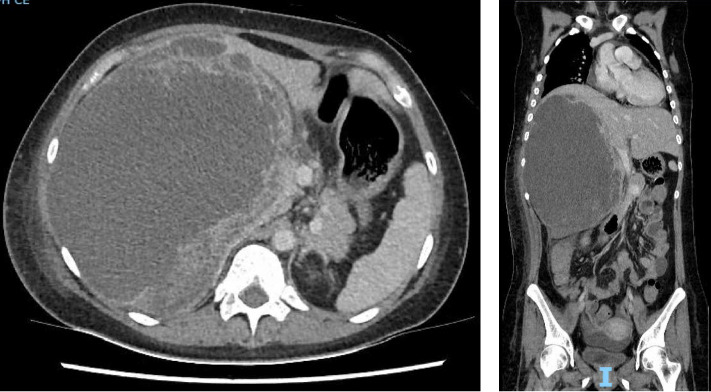
A very large cystic lesion in the right lobe of the liver measuring 15.2 × 17.3 × 21.7 cm with rim enhancement and multiple peripheral septations is seen on contrast-enhanced CT scan of the abdomen. (a) Transverse plane. (b) Coronal plane.

**Figure 2:**
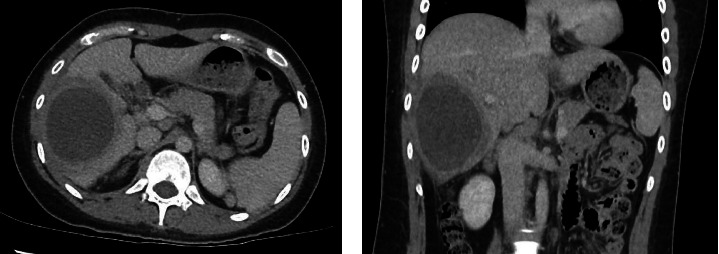
A repeat contrast-enhanced CT scan 11 weeks from the initial scan revealed a significantly smaller, unilocular hepatic lesion measuring 6.2 × 5.5 cm. The lesion had a smooth-enhancing rim surrounded by an outer rim of thick perilesional oedema, giving a ‘double-rim' appearance. (a) Transverse plane. (b) Coronal plane.

A 24-year-old female, with no significant medical history, presented to an emergency department in Singapore for 2 weeks of fever and right quadrant pain. The patient originated from Batam, Indonesia, where she lived with her family and worked as an administrative assistant. She was born and raised in Batam, Indonesia, and had not travelled to any other country apart from Singapore. She reported no known animal exposures. She did not drink directly from the tap, but obtained her drinking water from the local water refill station. She denied consumption of any raw or undercooked fish or crustaceans. On examination, she was febrile at 39.5 degrees Celsius and tachycardic. Her blood pressure was 98/58 mmHg. She was lethargic but nontoxic looking. Abdominal examination revealed marked hepatomegaly with right upper quadrant tenderness without rebound. Laboratory studies were remarkable for anaemia (haemoglobin 9.1 g/dL) and leucocytosis (white blood cell count 33.5 × 10^3^/μL). There was no eosinophilia. C-reactive protein and procalcitonin were elevated at 302.8 mg/L and 7.03 μg/L, respectively. Her liver function test was abnormal: total bilirubin (45.4 μmol/L), direct bilirubin (25.1 μmol/L), alkaline phosphatase (310 U/L), alanine transaminase (324 U/L) and aspartate transaminase (264 U/L). A contrast-enhanced computed tomography (CT) scan of her abdomen revealed a very large cystic lesion in the right lobe of the liver measuring 15.2 × 17.3 × 21.7 cm with rim enhancement and multiple peripheral septations (Figures 1(a) and 1(b)). Empirical intravenous ceftriaxone and metronidazole were initiated. Two sets of blood cultures, taken prior to antibiotic administration, eventually returned as no bacterial growth. The patient underwent percutaneous drainage of the liver lesion. Purulent fluid was aspirated. Gram stain, aerobic and anaerobic cultures of the liver aspirate were negative. No ova, cysts or parasites were seen on three consecutive stool microscopic examinations. The liver aspirate was sent to the National Public Health Laboratory (NPHL), a tertiary reference laboratory for further molecular diagnostic tests. DNA was extracted from 25 mg of liver aspirate using QIAamp® DNA Mini Kit according to manufacturer's instructions. *Entamoeba histolytica* PCR [2] and *Echinococcus* PCR [3] were performed. Only *E. histolytica* DNA was detected. Confirmation was obtained by amplification, sequencing and BLAST comparison of a large fragment of the 18S rRNA gene which showed 100% similarity with published *E. histolytica* sequences. The sequence has been deposited in GenBank under accession number OP925909. A diagnosis of ALA was made. The patient completed a course of metronidazole with good clinical response. She also received 1 week of paromomycin to eradicate intraluminal cysts. A repeat contrast-enhanced CT scan 11 weeks later revealed a significantly smaller, unilocular hepatic lesion measuring 6.2 × 5.5 cm (Figures 2(a) and 2(b)). The lesion had a smooth-enhancing rim surrounded by an outer rim of thick perilesional oedema, giving a ‘double-rim' appearance.

## 3. Discussion

Amoebiasis is caused by the protozoan *E. histolytica* whose life cycle consists of two stages: a trophozoite and a cyst form. It is transmitted via the faecal–oral route, through consumption of faecally contaminated food or water or exposure to faecal matter during sexual intercourse [4]. Therefore, the highest burden is in developing countries where there is poor sanitation. It is estimated that millions of people are infected with *E. histolytica*, and more than 55,000 deaths result from amoebiasis each year [5]. In developing countries, the exact burden of amoebiasis can be challenging to quantify due to factors such as limited diagnostic capacities and surveillance [6].

Indonesia is the fourth most populous country in the world, with a population of more than 270 million across 17,000 islands with varying population sizes and levels of sanitation. Batam is an island located at approximately 1°N, 104°E in the Indonesian province of Riau Islands with a population of more than 1.5 million people. There are limited published data on the burden of amoebiasis on Batam. A study in 2023 found that 26.6% (89/335) of the inhabitants of Sabang Island in rural Indonesia had stool samples that were positive on microscopy for *Entamoeba* species [7]. While the exact source of amoebiasis could not be conclusively determined in our patient based on her dietary history, it is probable that she acquired the infection through contaminated food or water given the burden of amoebiasis in Indonesia and the movement of the population between rural and urban Indonesia.

ALA is the most common form of extraintestinal amoebiasis and occurs when amoebae ascend through the portal venous system. However, ALA may present without concurrent colitis [8]. Previously thought to be a monomicrobial infection, recent studies suggest that ALAes may in fact be polymicrobial mixed abscesses (containing both bacteria and protozoa) [9]. Using partial 16S rRNA gene sequencing, Reyna-Fabian et al. identified several bacterial groups and *E. dispar* DNA, together with *E. histolytica* DNA, from liver abscess aspirate in what were clinically diagnosed as ALAes [9]. The authors hypothesised that as *E. histolytica* trophozoites translocate into the portal circulation, they carry along phagocytosed intestinal bacteria. The trophozoites and the bacteria seed the liver parenchyma, resulting in ‘mixed' liver abscesses [9]. Co-infecting intestinal bacteria may enable or enhance the virulence of *E. dispar,* challenging the traditional concept that *E. dispar* is nonpathogenic [9, 10].

ALA is more common in males than females [11, 12]. Patients present acutely or subacutely with fever and right upper quadrant pain [8, 13]. Laboratory studies show leucocytosis without eosinophilia and elevated alkaline phosphatase [8, 14, 15]. ALAes typically vary from a few centimetres to 16 cm [14–16]. Although classically a unilocular, well-defined lesion in the right hepatic lobe with rim enhancement and a peripheral zone of oedema (‘double rim' or ‘double target' sign) [16, 17], Priyadarshi et al. [13] recently classified the CT imaging features of ALAes into three distinct but overlapping patterns that correlate with different clinical subtypes, namely, acute aggressive (< 10 days), subacute mild (2 to 4 weeks) and chronic indolent (> 4 weeks). In acute aggressive ALA, imaging features include incomplete or absent wall, ragged edges, interrupted or no enhancement, septations, heterogeneous content and/or widespread or wedge-shaped perilesional hypodensity [13]. The ‘double-rim' sign appears only subsequently in the subacute phase [13]. In the chronic indolent phase, the wall no longer enhances and the perilesional oedema resolves, leading to the disappearance of the ‘double-rim' sign [13]. This varied morphology reflects the underlying pathological changes, which occur as ALA evolves through the different phases of maturation [13]. In our patient, the large septated abscess evolved to become a smaller unilocular lesion with a ‘double-rim' appearance after treatment (Figure 2).

The main differential diagnosis for ALA is PLA, and distinguishing between the two can be challenging. Blood cultures, if positive, support the diagnosis of a PLA. However, they are positive in only around 30% of PLAes [17], and the yield is further reduced by prior antibiotics. Because there is usually no concurrent colitis, the sensitivity of stool microscopy for the cysts or trophozoites in ALA is low [8]. The clinical and imaging features of PLAes and ALAes overlap considerably. The ‘double rim' sign may be present in both on contrast-enhanced CT [18]. The ‘cluster' sign, seen when multiple small low-attenuation lesions aggregate in a localised area and coalesce into a single larger abscess cavity, may be observed in multiloculated PLA but is not pathognomonic [18]. A Pakistani study of 577 patients with liver abscesses found that age > 50 years, pulmonary findings, multiple abscesses and amoebic serology titres < 1:256 IU were predictive of PLA [19]. Conversely, a Spanish study of 58 patients with liver abscesses found that age 45 years or younger, presence of diarrhoea and a solitary right lobar abscess favoured ALA [20]. Because neither symptomatology nor radiology is specific, ALA should be considered whenever there is epidemiological exposure. As the time from exposure to disease onset may be months to years [15], a comprehensive travel history, with particular attention to diet, is important so that appropriate microbiological testing can be performed. Serology testing is available but is less useful in long-term residents of endemic areas as a positive serology does not differentiate between current and previous infection. While there is no benefit of drainage in addition to medical therapy in uncomplicated ALAes [21], aspiration of the abscess should be considered when there is diagnostic uncertainty or when the abscess is large and at risk of rupture. In such cases, antigen or PCR testing on the liver aspirate can help diagnose ALA. Antigen detection using enzyme-linked immunosorbent assays (ELISAs) is available and has a shorter turnaround time compared to PCR-based assays, but reported sensitivity varies between studies and can be reduced by treatment [22, 23]. PCR-based assays are more sensitive than antigen-based tests [22–25] but may not be widely available due to lack of laboratory expertise and equipment. In Singapore, the PCR testing on the nonstool specimens, such as liver abscess aspirate, was only available at our NPHL. In view of the longer turnaround time for molecular diagnostic tests, empirical treatment should be initiated as soon as there is clinical suspicion, rather than be delayed until the diagnosis is confirmed. Trophozoites are seen in < 20% of aspirates and typically only seen when the wall is sampled [26], and hence cytology cannot be relied upon for diagnosis. Although our patient reported no exposure to canines, echinococcosis was considered as a differential diagnosis due to the large, cystic and septated appearance of the liver lesion. It is not uncommon to encounter hydatid cysts greater than 10 cm in diameter [27–29], and septations can be observed due to presence of daughter cysts. Fever is not typical in echinococcosis but can occur when there is bacterial superinfection or cyst rupture. Similar to amoebiasis, eosinophilia is often absent. There was initial concern for fluid spillage with seeding of protoscolices in the abdominal cavity during percutaneous drainage if the liver lesion was a hydatid cyst. Nevertheless, PLAes and ALAes were thought to be more likely, and thus percutaneous drainage was performed. In general, large abscesses that are more than 10 cm in diameter, whether pyogenic or amoebic, generally require drainage due to risk of rupture. However, the optimal approach, whether percutaneous catheter drainage versus surgical drainage, remains uncertain [21, 30, 31] but should be individualised based on microbiological diagnosis, patient factors and local expertise. Patients with ALAes generally respond well to metronidazole or tinidazole. A luminal agent should be subsequently administered to eradicate intraluminal cysts.

In conclusion, a comprehensive travel history is important when managing a patient with a liver abscess as the clinical and imaging features of PLAes and ALAes overlap considerably. Molecular diagnostic testing offers the highest sensitivity, but empiric treatment should be initiated as soon as possible while diagnostic evaluation is underway to prevent complications and death.

## Data Availability

Molecular data supporting the findings of this report have been deposited in GenBank under accession number OP925909; other data could be made available from the corresponding authors upon request.
